# AMF Community Diversity Promotes Grapevine Growth Parameters under High Black Foot Disease Pressure

**DOI:** 10.3390/jof8030250

**Published:** 2022-03-01

**Authors:** Romy Moukarzel, Hayley J. Ridgway, Jing Liu, Alexis Guerin-Laguette, E. Eirian Jones

**Affiliations:** 1 Faculty of Agriculture and Life Sciences, Lincoln University, Lincoln 7647, New Zealand; hayley.ridgway@plantandfood.co.nz (H.J.R.); jing.liu@plantandfood.co.nz (J.L.); eirian.jones@lincoln.ac.nz (E.E.J.); 2 The New Zealand Institute for Plant and Food Research Ltd., Private Bag 4704, Christchurch 8140, New Zealand; 3 Mycotree C/-Southern Woods Nursery, Christchurch 8441, New Zealand; alexis@mycotree.co.nz

**Keywords:** AMF community, *Ilyonectria*, *Dactylonectria*, disease severity, disease incidence, *Vitis* spp., rootstocks

## Abstract

Black foot disease is one of the main grapevine root diseases observed worldwide and is especially problematic in New Zealand. Arbuscular mycorrhizal fungi (AMF) have been shown to reduce infection and mitigate the effect of black foot disease on grapevine rootstocks. In contrast to prior studies, which have limited their focus to the effect of one, two or a combination of only a small number of AMF species, this study used whole AMF communities identified from 101-14, 5C and Schwarzmann rootstocks sampled from New Zealand vineyards. The effect of AMF on black foot disease was investigated in a ‘home’ and ‘away’ experiment using three commercial grapevine rootstocks. The study produced some evidence that AMF treatments lowered disease incidence at 5 cm and disease severity in vines by 40% to 50% compared to the vines inoculated with the pathogen only. This work also showed that the presence of high disease incidence may have limited the potential disease protective effect of AMF community. However, despite the high disease incidence and severity, AMF inoculation increased vine growth parameters by 60% to 80% compared to the vines inoculated with the pathogen only. This study is the first to provide an understanding on how young grapevine rootstocks inoculated with their ‘home’ and ‘away’ AMF communities would respond to challenge with a black foot pathogen species mixture. Further research is required to understand the mechanistic effect of AMF colonization on the increase of grapevine growth parameters under high black foot disease pressure.

## 1. Introduction

Cultivated grapevine varieties are susceptible to diseases caused by fungal pathogens which can limit yield. Historically, black foot disease has been commonly associated with soilborne *Cylindrocarpon* species, however based on the recent taxonomic revision of the genus, the species associated with black foot disease of grapevines have been reclassified as either *Dactylonectria* or *Ilyonectria*, with several species defined within each group [1,2,3]. Species from other soil-borne fungal genera including *Campylocarpon* spp., *Cylindrocladiella* spp., *Neonectria* spp. and *Thelonectria* spp. are also reported as associated with the disease [3,4,5,6]. The symptoms of black foot can be recognized by the development of black necrotic lesions on roots, and brown discoloration in the trunk base of the affected vines [6,7]. These pathogens can persist as resting spores or mycelium in infected root fragments in vineyard and nursery soils [6,8,9,10]. The black foot pathogens impact young grapevines during field establishment by infecting the root vascular tissues at the basal end of the rootstock contributing to poor growth or death of vines [11,12]. Moreover, the severity and spread of black foot disease is enhanced by environmental stress, and while management practices can improve the performance of diseased grapevines, there are no proven methods to control or fully eradicate the disease from infected vineyards [6]. 

Arbuscular mycorrhizal (AM) symbiosis is the most widespread type of interaction between plants and microbes in the context of phylogeny and ecology and they have been shown to provide protection against many fungal pathogens associated with grapevines [13]. They have also been shown to reduce infection and mitigate the effect of black foot disease on grapevine rootstocks [11]. Studies have shown that grapevine rootstock (*V. rupestris*) inoculated with *Glomus intraradices* prior to inoculation with ‘*Cylindrocarpon*’ *macrodidymum* (as *Dactylonectria macrodidyma* species complex) were less susceptible to black foot disease than non-mycorrhizal ones [11]. Another study showed that grapevine rootstocks inoculated with different AMF species were variable in their subsequent resistance to infection by ‘*Cylindrocarpon*’ spp. and that ‘*Glomus*’ *mosseae* (as *Funneliformis mosseae*) had a greater beneficial effect than *Acaulospora laevis* across all rootstocks [9]. It was also reported that pre-inoculating the vine with AMF shortly after rooting in the greenhouse or nursery, prior to transplanting into the field, could improve grapevine rootstock resistance to fungal pathogens [13]. However, in a recent study, inoculation with a commercial AMF inoculum containing *Rhizophagus irregularis* was shown to increase *Ilyonectria liriodendri* infection of grapevine rootstock Riparia Gloire when the AMF inoculant was applied either before or at the same time as pathogen exposure [14]. Further, AMF inoculation had no beneficial effect on any of the plant growth parameters assessed in that study. A recent study has shown that the genetic makeup of the host plant species can drive the AMF community recruitment in grapevines where the rootstocks sampled from the same site harbored different AMF communities [15]. Colonization with a diverse AMF community may promote vine growth and the uptake of nutrients [16] and can act as insurance to sustain plant development under changing environmental conditions [17]. Most of the aforementioned studies have only focused on the effects of individual AMF species on grapevine growth and black foot disease with no studies researching the effect of AMF communities as a whole on grapevine growth and black foot disease [9,11,14,18]. Therefore, the main objective of this study was to investigate whether the pre-inoculation of commercial grapevine rootstocks with their “home” or “away” AMF communities reduce black foot disease infection and symptom severity compared to non-AMF inoculated rootstocks. 

## 2. Materials and Methods

### 2.1. Origin and Maintenance of Fungal Pathogens

The pathogen isolates of *Ilyonectria liriodendri* (HB2d, Mar19f and WPa1e), *Ilyonectria europaea* (WPa1a) and *Dactylonectria macrodidyma* (Mar9b and CO6a) used in this study were obtained from the Lincoln University Plant Microbiology Culture Collection. These six isolates had been previously isolated from black foot disease symptomatic vines grown in the regions of Marlborough, Waipara and Central Otago and had been shown to be pathogenic in previous experimental work [8,19]. These isolates were stored as mycelial discs in glycerol at −80 °C and were routinely cultured on potato dextrose agar (PDA; BD Difco™) at 20 °C in the dark for 14 days.

### 2.2. Propagation Material and AMF Inoculum

Dormant stem cuttings of the three rootstocks (101-14, 5C and Schwarzmann) used for this experiment were obtained from Riversun Nursery (Gisborne, New Zealand). The AMF inoculum used in this study for each rootstock had previously been isolated and identified from harvested pot cultures as described in Moukarzel et al. (2021) [17]. The 101-14 AMF spore inoculum was dominated by *Funneliformis* sp. Followed by *Glomus* sp. 2, *Glomus* sp. 1 and *Ambispora* sp. The 5C AMF spore inoculum was dominated by *Ambispora* sp. followed by *Funneliformis* sp., *Glomus* sp. 1 and *Glomus* sp. 2. Schwarzmann AMF spore inoculum was dominated by *Claroideoglomus* sp. followed by *Glomus* sp. 1, *Glomus* sp. 2 and *Funneliformis* sp. 

### 2.3. Experimental Design

The experiment was conducted in a greenhouse at Lincoln University, New Zealand (43.6434° S, 172.4678° E). The three rootstock varieties (101-14, 5C and Schwarzmann) were pre-colonized, or not, with AMF and then inoculated with a mixed inoculum of six *Dactylonectria*/*Ilyonectria* spp. isolates. Each of the commercial rootstocks were inoculated with their own AMF communities and the AMF communities of each of the other rootstocks two months prior to inoculation with the pathogens. Control treatments with no AMF and no pathogen were also included in the design. The treatments are described in Table 1. Each treatment was applied to 10 potted rootstocks, and the treatments were arranged in a completely randomized block design on metal wire tables in the greenhouse.

### 2.4. AMF Treatment Application

Four large plastic containers (713 mm L × 442 mm W × 222 mm H) were filled with sterilized damp pumice and either mixed with the specific AMF community inoculum or left uninoculated depending on the treatment. Container 1 had no AMF inoculation (Ctrl/Ctrl and Ctrl/pathogen treatments in Table 1), and containers 2, 3 and 4 had 101-14, 5C and Schwarzmann AMF communities (AMF/Ctrl and AMF/pathogen treatments), respectively. The pumice in each container was inoculated with approximately 10,000 spores (5 spores/g) at the corresponding AMF community diversity and relative abundance for each rootstock. The spores were added and mixed thoroughly with the pumice in each of the containers. The stems, cut into two-bud cuttings, were dipped in powdered rooting hormone (Dynaroot 3 plant growth regulator; active ingredient β-indolulbutyric acid, 8 g/kg) and planted at a depth of 15 cm in the pumice. The containers were planted with 25 cuttings of each rootstock at the beginning of November 2019 and were placed on heating pads to stimulate root development for six weeks in a shade house.

### 2.5. Pathogen Inoculum Preparation 

The spore inoculum of the *Dactylonectria* and *Ilyonectria* spp. isolates was prepared from 14-day old colonies growing on PDA. Colonies were flooded with approximately 10 mL tap water amended with three drops/L of Tween 80 (polyoxylethylene (20) sorbitan mono-oleate; BDH Chemicals Ltd., Poole, England) and the surface of the colonies were scraped with the edge of a sterile glass slide (new slide used for each isolate) as described by Probst et al. (2019) [8]. Conidial concentration in the resulting suspensions were determined based on hemocytometer counts. Due to the uneven spore production by the different isolates, the spore suspensions of *I. liriodendri* isolates (HB2d, Mar19f and WPa1e), which produced many spores, were adjusted to 1 × 10^6^ spores/mL. The two *D. macrodidyma* isolates (Mar9b, CO6a) and the *I. europaea* isolate (WPa1a), which had lower spore production, were adjusted to 1 × 10^4^ spores/mL. The mixed isolate conidial suspension contained 25 mL of 1 × 10^4^ spores/mL of each of Mar9b, CO6a, WPa1a and 650 mL of 1 × 10^6^ spores/mL each of HB2d, Mar19f and WPa1e. The final mixed isolate spore suspension concentration was 9.6 × 10^5^ spores/mL. 

### 2.6. Pathogen Treatment Application

In mid-December 2019, the rooted grapevine rootstocks (AMF and non-AMF treated) were inoculated with *Dactylonectria*/*Ilyonectria* spp. by soaking the rootstocks in a mixed isolate conidial suspension in 10 L plastic buckets. Four buckets were filled with 2025 mL tap water and used to soak the non-pathogen controls; the other four buckets contained 2025 mL of mixed isolate conidial suspension. One bucket was used for each of the AMF treatments (four for pathogen inoculated and four for non-pathogen inoculated). The tips of the roots of the rootstocks were lightly trimmed using an ethanol sterilized scissors (70% ethanol between each treatment) and 10 grapevine rooted cuttings of the three cultivars treated with the same AMF treatment were soaked in the same bucket (i.e., 101-14, 5C and Schwartzman rooted cuttings treated with 101-14 AMF). The cuttings were soaked for 30 min either in the spore suspension or in tap water, before being potted up into black polyethylene bags (approx. 3 L capacity: 120 mm L × 120 mm W × 200 mm H). The pots were half filled with potting mix (50% sterile silica sand, 40% pumice and 10% low phosphorus potting mixture containing fertilizers: horticultural bark, Osmocote 38-0-0, Osmocote 0-0-32, horticultural lime, Micromax trace elements and Hydraflo), with all products being manufactured by Everris International, Geldermalsen, The Netherlands and purchased from Intelligro, New Zealand. The cuttings were placed into the pots and then the pots filled with potting mix. The plants were watered lightly, shoots trimmed to two nodes and any flowers were removed. The pots were left overnight in the potting room to acclimatize before being placed in their randomized block design on metal wire tables in the greenhouse. To ensure infection, the pathogen treatment was also applied in mid-March 2020. For this inoculation, a mixed isolate conidial suspension (9.6 × 10^5^ spores/mL) produced as described previously was used. The rootstock root system was wounded using a sharp knife driven vertically into the potting mix at four equidistance positions about 2.5 cm from the trunk base and each rootstock was inoculated with 50 mL of the conidial suspension followed by 50 mL water to ensure that the spores were dispersed to the root zone as described by Brown et al. (2013) [20]. 

### 2.7. AMF Colonization Confirmation 

For the remaining five rootstocks per variety and AMF treatment the roots were removed at the end of setting up the experiment and placed in separate tubes and stored in a cool box until the following day. A representative sample (0.2 g) was taken from each root sample and used for confirmation of AMF colonization using the staining method described in Moukarzel et al. (2020) [21]. 

### 2.8. Harvesting Process

In May 2020, six months after inoculating the grapevine cuttings with AMF, the plants were removed from the plastic bags. The shoots were cut from the stem and were placed in labelled paper bag, dried in an oven at 60 °C for 48 h and then weighed. The roots of each plant were thoroughly washed and then cut at the base of the stem and were placed in labelled paper bags and weighed after being dried in an oven at 60 °C for 48 h. The stem of each grapevine plant was placed in separate paper bags, stored at 4 °C and used for re-isolation of the pathogen to determine disease incidence and severity.

### 2.9. Pathogen Incidence and Severity Assessments

The lower sections of the rootstocks of all potted grapevine plants were cut to a length of six cm. The stems were surface sterilized using the method described by Holland et al. (2019) [14], whereby the stems were submerged in 70% ethanol for 30 s and then passed through a flame for 10 s. The stems were left to dry for 5 min in a sterile airflow in a laminar flow cabinet. The lowest 10 mm of the stem comprising the root crown was discarded and a 1–2 mm piece was sliced from the basal end of the stem (0 cm), cut into four pieces, and placed equidistantly near the edge of a Petri dish containing PDA amended with 250 mg/L chloramphenicol. A 1–2 mm transverse stem piece was also sliced at 5 cm above the base and plated in the center of the same plate. The Petri dishes were then sealed and incubated for seven days at 20 °C in the dark. The plates were regularly monitored for the growth of *Ilyonectria* and *Dactylonectria* spp. colonies from the wood pieces and were used to confirm the presence of black foot disease by comparing the colony morphology and conidia with the cultures of the three species used for inoculation. The presence of the pathogen in the grapevine stems at 0 cm or 5 cm (disease incidence) and the proportion of wood pieces at 0 cm colonized by the pathogens (disease severity) were recorded [8].

The presence/absence of other fungal groups isolated from the wood pieces for each plate was also recorded. These fungi were sub-cultured for morphological and molecular identification (Figure S1, Table S1). These data were used to determine whether AMF also reduced colonization by other potential pathogens or increased beneficial endophytes such as *Trichoderma* spp. 

### 2.10. Molecular Confirmation of Pathogen Identity

The identity of representative colonies (10% of each treatment) recovered from the grapevine stem pieces from the different treatments and presumptively identified as *Dactylonectria* and *Ilyonectria* was confirmed by sequencing a portion of the histone H3 gene region [1,2,3]. Genomic DNA was extracted from mycelium from the recovered colonies and from pure cultures of the *D. macrodidyma*, *I. europaea* and *I. liriodendri* isolates used for inoculation. From each colony, the mycelium was scraped using a 200 µL tip and added into a 1.7 mL tube containing 500 µL of 10% Chelex^®^ 100 Chelating Resin (cation exchange resin, sodium form, 1% cross-linkage, 100–200 dry mesh size, 150–300 µm wet bead size, BIO-RAD). Each tube was vortexed for 10 s and placed on a heating block for 10 min at 100 °C. The tubes were removed, and the pressure was released by opening each tube. Then the tubes were vortexed and placed back on the heating block as previously described. The tubes were centrifuged for 10 min at 13,000 r.p.m. and the supernatant (~150 µL) was removed and placed into a new 1.7 mL tube. The DNA concentration for each sample was measured using a Thermo Scientific™ Nanodrop Lite Spectrophotometer Nanodrop (Auckland, New Zealand). Each sample was adjusted using Millipore water to a final concentration of 30–50 ng/µL before PCR amplification. 

Sequencing of a portion of the *HIS* gene region was performed after PCR amplification using 0.2 mM dNTPs, 10 pmol of each primer, 5 U of Taq DNA polymerase and the supplied reaction buffer (Promega Inc., Seoul, Korea) in a total volume of 25 µL as follows: 94 °C for 5 min, followed by 40 cycles at 94 °C for 30 s, 52 °C for 30 s and 72 °C for 80 s, and a final elongation at 72 °C for 10 min. Primers were CYLH3F (5′-AGG TCC ACT GGT GGC AAG-3′) and CYLH3R (5′-AGC TGG ATG TCC TTG GAC TG-3′) for *HIS* [22]. After confirmation of successful amplification by agarose gel electrophoresis, amplicons were sequenced in both directions with the corresponding PCR primers using Sanger sequencing at the Lincoln University sequencing facility. Sequences were assembled and edited to resolve ambiguities using the BioEdit. Each sequence was compared by basic local alignment search tool (BLAST) to those on NCBI to identify similar sequences. The sequenced amplicons were also compared with the sequences of the inoculated isolates.

### 2.11. Statistical Analysis 

All statistical analyses were conducted as appropriate for a completely randomized design using R studio. Differences in vine growth responses were determined between the treatments using a one-way analysis of variance (ANOVA). Differences between the treatment means were detected using a general linear hypothesis within the multcomp R-package. Black foot disease incidence data were analyzed using the generalized linear model (GLM) to compare disease incidence with significance level used *p* = 0.05. 

## 3. Results

### 3.1. AMF Colonization Confirmation in Roots

**Figure 1:**
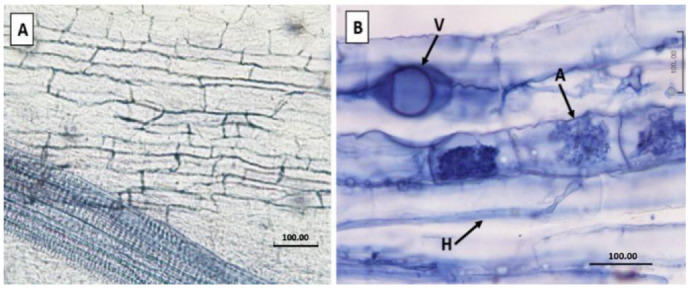
AMF colonization confirmation using Trypan blue stain. (**A**): Grapevine roots of 101-14 rootstock from the uninoculated AMF control. (**B**): Grapevine roots of 101-14 rootstock pre-colonized with 101-14 AMF community. V: vesicle, A: arbuscules, H: hypha. Scale bar represents 100 µm.

None of the grapevine root samples from the three rootstock varieties rooted in the uninoculated pumice (no AMF treatments) were colonized with AMF, with no visible AMF structures observed in the root material (Figure 1A). For the rootstocks rooted in AMF inoculated pumice, AMF colonization was confirmed by the presence of vesicles, arbuscules and hyphae (Figure 1B) in all rootstocks for all AMF treatments confirming that the roots of the different grapevine rootstocks were pre-colonized with ‘home’ and ‘away’ AMF communities.

### 3.2. Plant Growth Parameters at Harvest

#### 3.2.1. Shoot Dry Weight

**Figure 2:**
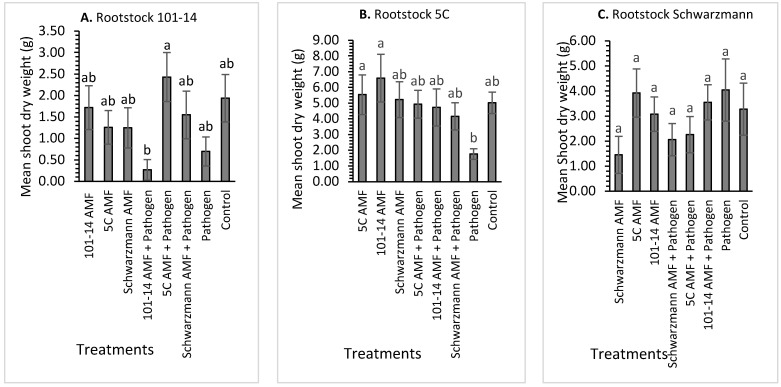
Mean shoot dry weight (g) at harvest for rootstocks 101-14 (**A**), 5C (**B**) and Schwarzmann (**C**) colonized with different AMF communities originating from different rootstocks (101-14, 5C and Schwarzmann) and inoculated with black foot pathogen isolates. Bars with different letters are significantly different (*p* ≤ 0.05) based on the SED of mean comparisons generated from one-way ANOVA analysis in R. Error bars show ± SED.

For rootstock 101-14, there was a significant effect (*p* = 0.048) of treatment on shoot dry weight, with the mean shoot dry weight varying between 0.35 g for vines inoculated with the 101-14 and pathogen and 2.50 g for vines inoculated with the ‘away’ 5C derived AMF community and the pathogen (Figure 2A). The shoot dry weight of vines inoculated with the ‘away’ 5C derived AMF community and pathogen were significantly higher by 86% compared with vines inoculated with the ‘home’ 101-14 derived AMF community and pathogen. 

For rootstock 5C, there was a significant effect (*p* = 0.016) of treatment on shoot dry weight with the mean shoot dry weight (g) varying between 2.00 g for vines inoculated with the pathogen and 6.80 g for vines inoculated with the ‘away’ 101-14 derived AMF community (Figure 2B). The shoot dry weight of rootstock 5C inoculated with the ‘home’ 5C derived and ‘away’ 101-14 derived AMF communities was significantly higher, with an increase of 65% and 70%, respectively, in shoot dry weight, compared to 5C vines inoculated with the pathogen only. None of the other treatments differed significantly from each other. 

For rootstock Schwarzmann, treatment had no significant effect (*p* = 0.330) on shoot dry weight, with the mean shoot dry weight (g) varying between 1.20 g for vines inoculated with the ‘home’ Schwarzmann derived AMF community and 4.00 g for vines inoculated with 5C AMF and pathogen only (Figure 2C).

#### 3.2.2. Root Dry Weight

**Figure 3:**
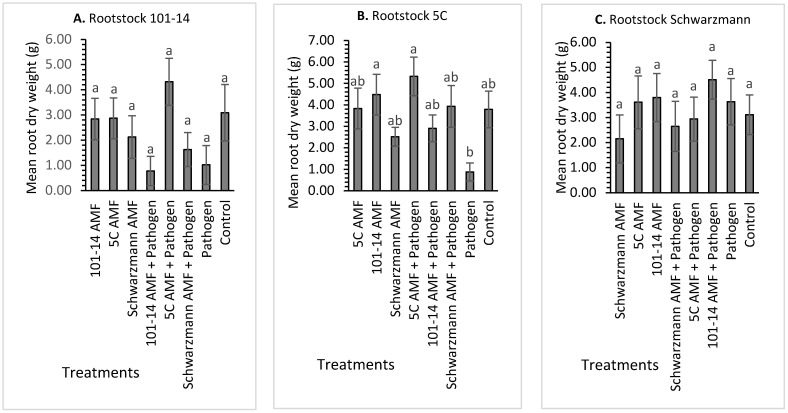
Mean root dry weight (g) at harvest for rootstocks 101-14 (**A**), 5C (**B**) and Schwarzmann (**C**) colonized with different AMF communities originating from different rootstocks (101-14, 5C and Schwarzmann) and inoculated with black foot pathogen isolates. Bars with different letters are significantly different (*p* ≤ 0.05) based on the SED of mean comparisons generated from one-way ANOVA analysis in R. Error bars show ± SED.

For rootstock 101-14, there was no significant effect (*p* = 0.067) of treatment on root dry weight, with the mean root dry weight (g) varying between 0.1 g for 101-14 vines inoculated with the ‘home’ 101-14 derived AMF community and pathogen and 4.5 g for vines inoculated with the ‘away’ 5C derived AMF community and pathogen (Figure 3A). For rootstock 5C, there was a significant effect (*p* = 0.007) of treatment on root dry weight with the mean root dry weight (g) varying between 0.8 g for vines inoculated with the pathogen and 5.8 g for vines inoculated with the ‘home’ 5C derived AMF community and pathogen (Figure 3B). The root dry weight of rootstock 5C inoculated with the ‘away’ 101-14 derived AMF community and ‘home’ 5C derived AMF community and pathogen was significantly higher, with an increase of 60% and 70%, respectively, in root dry weight, compared to 5C vines inoculated with the pathogen only. None of the other treatments differed significantly from each other. As for rootstock Schwarzmann, treatment had no significant effect (*p* = 0.514) on root dry weight, with the mean root dry weight (g) varying between 2.2 g for vines inoculated with the ‘home’ Schwarzmann derived AMF community and 4.4 g for vines inoculated with ‘away’ 101-14 derived AMF community and pathogen (Figure 3C).

### 3.3. Disease Incidence

Disease symptoms were observed on the vines 20 weeks after pathogen inoculation. The symptoms observed included roots with dark brown soft areas and brown discoloration at the base of the rootstock trunk. The overall statistical test on the main factors showed that there was a significant (*p* < 0.001) main effect of rootstock where 101-14 (33.75% ± 3.18) was significantly less affected by the pathogen compared to 5C (48.12% ± 3.97) and Schwarzmann (57.81% ± 4.3). The statistical test also showed a significant (*p* < 0.001) interaction between the rootstock and the pathogen inoculation treatments. Additionally, there were significant differences (*p* < 0.001) between the pathogen inoculated treatments and pathogen uninoculated treatments in the three rootstocks where the disease incidence in the 101-14 pathogen inoculated treatment (46.87% ± 4.83) were significantly higher compared to 101-14 pathogen uninoculated treatments (20.62% ± 2.95). For rootstock 5C, the pathogen inoculated treatments (63.75% ± 5.05) were significantly higher compared to the pathogen uninoculated treatments (32.5% ± 5.07). 

**Figure 4:**
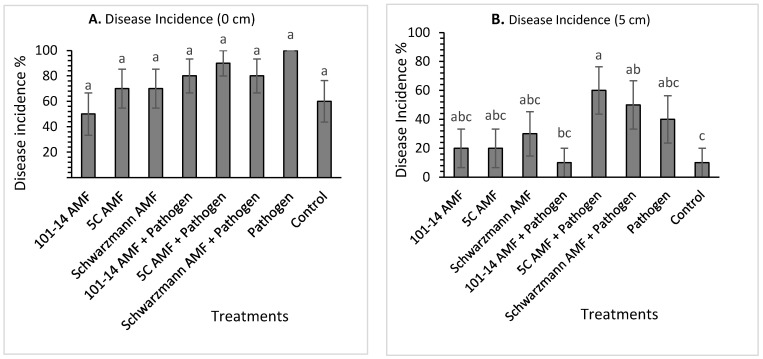
Mean black foot disease incidence (%) for rootstock 101-14 at 0 cm (**A**) and 5 cm (**B**) from the stem base colonized with different AMF communities originating from different rootstocks (101-14, 5C and Schwarzmann) and inoculated with black foot pathogen isolates. Bars with different letters are significantly different (*p* ≤ 0.05) based on the SED generated from GLM analysis. Error bars show ± SED.

For rootstock 101-14, there was no significant effect (*p* = 0.218) of treatment on disease incidence at 0 cm above the stem base. The disease incidence varied between 50% for vines pre-inoculated with the ‘home’ 101-14 derived AMF and 100% for vines inoculated with the pathogen only (Figure 4A). There was a significant effect (*p* = 0.044) of treatment on the disease incidence at 5 cm above the stem bases (Figure 4B). Vines inoculated with the ‘away’ 5C derived AMF community and pathogen had significantly greater mean disease incidence than vines inoculated with the ‘home’ 101-14 derived AMF community and pathogen and the uninoculated control treatments, with disease incidence being 60% compared with 10% for both the ‘home’ 101-14 derived AMF community and pathogen, and the uninoculated control treated vines. Moreover, vines inoculated with the ‘away’ Schwarzmann derived AMF community and pathogen had significantly greater mean disease incidence than the uninoculated control treatment, with disease incidence being 50% compared with 10% for the control. The other treatments did not show any significant differences. 

**Figure 5:**
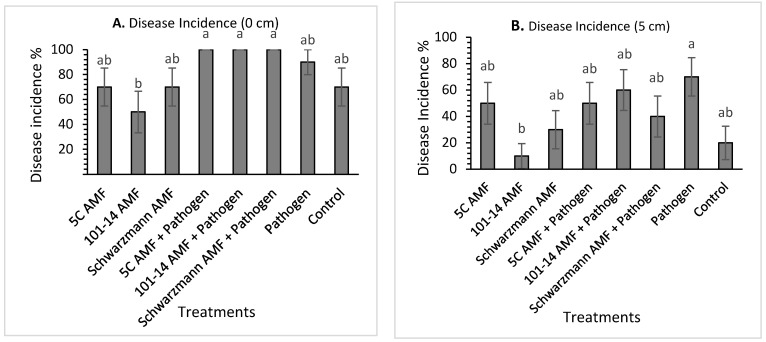
C at 0 cm (**A**) and 5 cm (**B**) from stem base colonized with different AMF communities originating from different rootstocks (101-14, 5C and Schwarzmann) and inoculated with black foot pathogen isolates. Bars with different letters are significantly different (*p* ≤ 0.05) based on the SED generated from GLM analysis. Error bars show ± SED.

For rootstock 5C, there was a significant effect (*p* = 0.017) of treatment on disease incidence at 0 cm above the stem base (Figure 5A). The disease incidence varied between 50% for vines pre-inoculated with the ‘away’ 101-14 derived AMF community and 100% for vines inoculated with the ‘home’ and ‘away’ AMF and pathogen treatments. Disease incidence was significantly higher (by 50%) for vines inoculated with ‘home’ and ‘away’ AMF and pathogen compared with the ‘home’ 101-14 derived AMF community. All the other treatments did not differ in their mean disease incidence (Figure 5A). Disease incidences at 5 cm above the stem base was significantly affected (*p* = 0.031) by treatment (Figure 5B). Disease incidence was significantly higher in the pathogen only treatment compared with vines inoculated with the ‘away’ 101-14 derived AMF community, with disease incidence being 70% compared with 10% for the ‘away’ 101-14 derived AMF community treatment. The other treatments were not significantly different (Figure 5B).

**Figure 6:**
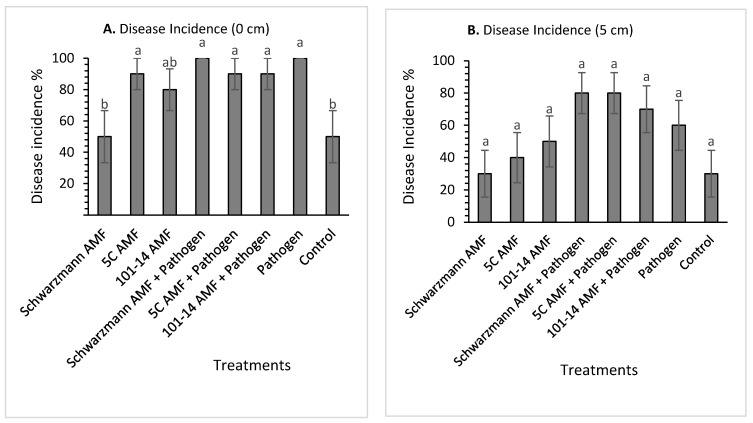
Mean black foot disease incidence (%) for rootstock Schwarzmann at 0 cm (**A**) and 5 cm (**B**) from stem base colonized with different AMF communities originating from different rootstocks (101-14, 5C and Schwarzmann) and inoculated with black foot pathogen isolates. Bars with different letters are significantly different (*p* ≤ 0.05) based on the SED generated from GLM analysis. Error bars show ± SED.

For rootstock Schwarzmann, there was a significant effect (*p* = 0.005) of treatment on disease incidence at 0 cm above stem base (Figure 6A). The disease incidence varied between 50% for vines inoculated with the ‘home’ Schwarzmann derived AMF community and the control and 100% for vines inoculated with the ‘home’ Schwarzmann derived AMF community and pathogen and the pathogen only treatment. Disease incidence was significantly higher in vines inoculated with the pathogen only, ‘away’ 5C derived AMF, ‘away’ 5C derived AMF and pathogen, ‘away’ 101-14 derived AMF and pathogen and ‘home’ Schwarzmann derived AMF and pathogen, with disease incidence being 90% and 100% compared 50% for vines inoculated with the ‘home’ Schwarzmann derived AMF community and the control. None of the other treatments differed significantly. Disease incidences at 5 cm above the stem base did not differ significantly between treatments (*p* = 0.093) (Figure 6B).

### 3.4. Disease Severity

**Figure 7:**
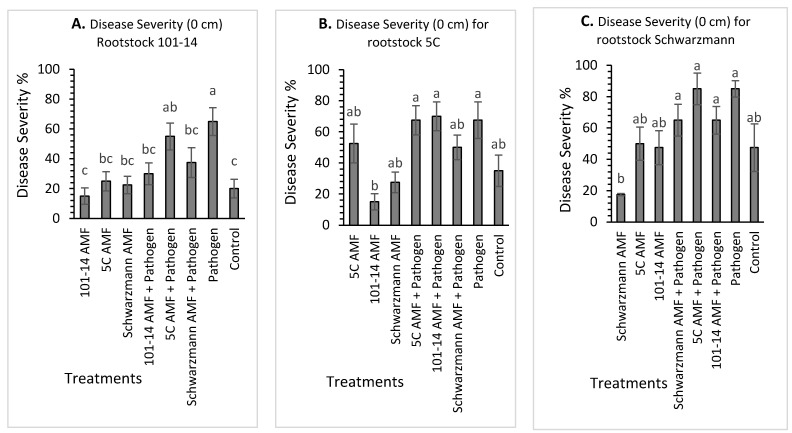
Mean black foot disease severity (%) for rootstocks 101-14 (**A**), 5C (**B**) and Schwarzmann (**C**) at 0 cm from stem base colonized with different AMF communities originating from different rootstocks (101-14, 5C and Schwarzmann) and inoculated with black foot pathogen isolates. Bars with different letters are significantly different (*p* ≤ 0.05) based on the SED generated from GLM analysis. Error bars show ± SED.

For rootstock 101-14, there was a significant effect (*p* < 0.001) of treatment on disease severity at 0 cm above the stem base. The disease severity varied between 15% for vines pre-inoculated with the ‘home’ 101-14 derived AMF community and 65% for vines inoculated with the pathogen only. The vines inoculated with the pathogen only had significantly greater mean disease incidence compared with the untreated control treatment and all ‘home’ and ‘away’ AMF treatments with or without pathogen, except for the ‘away’ 5C derived AMF community with pathogen treatment, with disease severity being 65% for the pathogen only treatment compared with 15% to 37% for the other treatments (Figure 7A). Disease severity was also significantly higher in vines inoculated with the ‘away’ 5C derived AMF community and pathogen treatment (55%) than the ‘home’ 101-14 derived AMF community (16%) and the control treatment (20%).

For rootstock 5C, there was a significant effect (*p* < 0.001) of treatment on disease severity at 0 cm above stem base (Figure 7B). The disease severity varied between 15% for vines pre-inoculated with the ‘away’ 101-14 derived AMF community and 70% for vines inoculated with the ‘away’ 101-14 derived AMF community and pathogen. Disease severity was significantly higher for vines inoculated with ‘away’ 101-14 derived AMF community and pathogen, ‘home’ 5C derived AMF community and pathogen, and pathogen only than vines inoculated with ‘home’ 101-14 derived AMF community, with disease severity being 70.0%, 67.5% and 67.5%, respectively, compared with 15.0% for the ‘home’ 101-14 derived AMF community. All the other treatments did not differ in their mean disease severity (Figure 7B).

For rootstock Schwarzmann, there was a significant effect (*p* < 0.001) of treatment on disease severity at 0 cm above stem base (Figure 7C). The disease severity varied between 17.5% for vines inoculated with the ‘home’ Schwarzmann derived AMF community and 85% for vines inoculated with the pathogen only. Disease severity was significantly higher in vines inoculated with the pathogen only, the ‘away’ 5C derived AMF community and pathogen, the ‘away’ 101-14 derived AMF community and pathogen and ‘home’ Schwarzmann derived AMF community and pathogen compared with vines inoculated with the ‘home’ Schwarzmann derived AMF community, with disease severity being 85.0%, 82.5%, 65.0% and 65.0%, respectively, compared with 17.5% for the ‘home’ Schwarzmann derived AMF community treatment. None of the other treatments differed significantly.

### 3.5. Black Foot Disease Confirmation

The identity of representative isolates recovered from the stem pieces for the different treatments and morphologically identified as *Dactylonectria* or *Ilyonectria* was confirmed by sequencing of the histone H3 gene region. The sequences confirmed the identity of the isolates as *Dactylonectria* or *Ilyonectria* species, confirming infection with black foot disease. Isolates recovered from the AMF/Pathogen and Pathogen treated vines were identified as *Ilyonectria liriodendri, Dactylonectria macrodidyma* and *D. torrensis* (Table S2). Moreover, isolates recovered from the AMF and control treatments, which were not inoculated with black foot pathogen isolates were identified as *Ilyonectria* sp., *I. liriodendri*, *D. torrensis*, *D. macrodidyma* and *D. novozelandica*.

## 4. Discussion

This research represents the first attempt to elucidate the effects of the whole AMF community on black foot disease infection and growth parameters in grapevines. This contrasts with other studies which have only focused on the effects of specific AMF species on disease levels [9,11,14]. Additionally, prior studies used commercial AMF inoculum that originated from other crops or species isolated from other crops, while in the present study the AMF communities originated from grapevines. Although AMF are considered general symbionts, distinctive AMF communities are associated with specific hosts in coexisting plant species such as grasses [23,24,25], forbs [26] and trees [27,28]. Even plants of the same species that differed in age harbored distinctive AMF [29,30] which was also observed in Moukarzel et al. (2021) [15] where different rootstocks from the same site had different AMF communities that are beneficial for their growth and development. Therefore, this study provides new understanding on how young grapevine rootstocks pre-inoculated with their ‘home’ and ‘away’ AMF communities respond to challenge with black foot pathogens. 

Inoculation with the different AMF communities did not reduce disease incidence or severity compared with the uninoculated (non-pathogen) control. One possible explanation for this is that as the sites the communities were obtained from did not have high black foot disease there was no driver to accumulate black foot protective AMF taxa. There are several mechanisms for how AMF protect plants against pathogens. It is reported that AMF may reduce pathogen infection by activating defense response mechanism in host plants [31,32,33] also known as mycorrhizal induced systemic resistance (MIR). Studies reported that the ability of AMF to induce pathogen susceptibility is linked to the timing of AMF inoculation [14,18,34]. It is suggested that grapevine AMF inoculation should occur prior to pathogen exposure which is necessary to activate the defense mechanism [34]. Holland et al. (2019) [14] commented that inoculating young grapevine with AMF at the nursery could potentially reduce black foot disease infection. This could explain why colonization by AMF did not have any effect on the ‘resident’ infection levels in the rootstock cultivars. A study showed that infection by other fungi is limited when AMF colonize roots [34]. This indicates that AMF colonization in the roots provide a barrier against pathogen infection via competition for space [31,34,35]. In Schwarzmann vines the disease incidence at 0 cm was significantly higher for vines inoculated with AMF derived from 5C only compared with the uninoculated vines which suggest that specific AMF taxa and their combination could have specific effect on host plant. A study showed that *Rhizoctonia solani* was significantly reduced in mixture containing *Rhizophagus clarus* while other AMF species such as *R. intraradices* and *Claroideogloms etunicatum* did not alleviate the effects of the pathogen individually, however, when combined, a significant increase in dry shoot biomass was observed in comparison to the pathogen control [36]. 

The lack of significant disease control seen in this study might, in part, be due to the high disease pressure in the pathogen inoculated treatments, with disease level (incidence at 0 cm above the stem base) being 100% in several treatments for the different rootstocks. A biological agent such as AMF may struggle to reduce disease infection at such high disease pressure. This was observed in the Bleach et al. (2008) study where a reduction in disease occurred at medium disease pressure (<25%) but not at high disease pressure levels (>50%) in field planted vines. A reduction in disease level was observed in other studies where *Glomus* sp. reduced *Fusarium oxysporum* disease levels from 51% to 24.6% in tomato [37] and *Erysiphe pisi* disease levels from 55.2% to 28.7% [38] in pea plants. In another study, the control of *Sclerotinia sclerotiorum* using *Coniothyrium minitans* as a biocontrol agent was only seen when disease levels were less than 40% [39]. 

The inoculation method used in this study might have caused high disease level. The high disease level was not anticipated as Probst et al. (2019) showed inoculation of wounded roots by soaking in spore suspension of similar concentration with the pathogens *I. liriodendri* or *D. macrodidyma* resulted in lower disease. In another study, where *Vitis rupestris* were inoculated with 6 mL of spore suspension (10^7^ conidia/mL) directly into the pot, mycorrhizal plants showed significantly lower disease severity [40]. In this study the high disease level was probably because the vines in this experiment were also inoculated by wounding the roots and applying the inoculum as a soil drench three months later. This also reflects the variability of biological systems when working with both plants and fungi where minor changes in any of the variables may result in higher or lower disease incidence. In future inoculating with lower inoculum concentration may be prudent since Probst et al. (2019) [8] showed that as little as 10^2^/mL conidia resulted in disease.

Although there was no dominant effect of inoculation of the three rootstocks with the different AMF communities on disease levels in this study, there was slight evidence that some of the rootstock/AMF community combinations decreased black foot disease incidence and severity. This was observed in rootstock 101-14 inoculated with its ‘home’ 101-14 AMF community which showed lower disease incidence at 5 cm and disease severity. The same effect on disease severity was observed when 101-14 was inoculated with ‘away’ Schwarzmann AMF compared with the pathogen control. There is also an indication (trend) that rootstock 5C inoculated with the ‘away’ Schwarzmann AMF community had lower disease incidence at 5 cm and overall disease severity. Similar results were observed in greenhouse experiments where AMF decreased infection by the root pathogens *Phytophthora parasitica* [41] and *Fusarium oxysporum* [42] in papaya and banana, respectively. Moreover, in the Petit and Gubler (2006) [40] study, it was shown that St. George (*Vitis rupestris*) vines inoculated with *Rhizophagus intraradices* (as *G. intraradices*) were less susceptible to black foot disease caused by *C. macrodidymum* than non-mycorrhizal vines. They suggest that decreased susceptibility was through AMF enhanced plant resistance to biotic stresses. However, this was not the case for the two other rootstocks in the current study, where pre-inoculation with ‘home’ and ‘away’ AMF communities increased disease incidence and severity in Schwarzmann and 5C vines, suggesting that disease resistance is a result of specific AMF community/rootstock interactions. Increased disease was also observed in a recent Canadian study where AMF inoculation increased the incidence of *Ilyonectria* sp. in grapevine rootstock Riparia Gloire [14]. Furthermore, in the Bleach et al. (2008) [9] study, there was no black foot infection in rootstocks Riparia Gloire and 3309C inoculated with *Funneliformis mosseae* (as *G. mosseae*). This could indicate that different rootstocks interact differently and receive different benefits from specific AMF species within the selected communities that colonize their roots. 

There was some evidence that for rootstocks 101-14 and 5C that pathogen-associated reductions in growth could be partly reversed by some of the AMF treatments. This suggests that, even though the plants are infected by the pathogens, that the AMF benefited them in some cases by helping them recover, indicating a more important role in resilience to infection than in resistance to infection. In the presence of the pathogen, 101-14 vines performed better with respect to plant growth parameters when pre-inoculated with ‘away’ 5C AMF community than with their ‘home’ 101-14 derived AMF community, having higher root and shoot dry weight. A similar trend was also seen for 5C vines were most of the growth parameters were higher in vines pre-inoculated with AMF community originally derived from 101-14 with or without the pathogen compared with the ‘home’ 5C derived AMF community. One explanation for this could be related to AMF taxa as it seems that 101-14 could benefit more from *Glomus* spp. that are present in higher abundance in AMF inoculum derived from 5C rootstock but not present in AMF inoculum derived from the 101-14 and Schwarzmann rootstocks. Similarly, 5C rootstock seemed to benefit more from higher abundance of *Funneliformis mosseae* that was available in the inoculum derived from 101-14 but not in the other AMF communities derived from 5C and Schwarzmann rootstocks. A similar trend was observed in the Bleach et al. (2008) [9] study where rootstocks responded differently to AMF inoculation with the variation in responses depending on specific AMF and rootstock combination. In the same study it was observed that inoculating rootstocks 5C, Riparia Gloire and Schwarzmann with *F. mosseae* (as *‘G.’mosseae*) increased shoot dry weight while when these rootstocks were inoculated with *A. laevis,* the effect was different as shoot dry weight deceased. 

In some cases, rootstocks inoculated only with the AMF communities had lower shoot and root dry weight compared to rootstocks that were inoculated with both AMF and the pathogen. As suggested by Holland et al. (2019) [14], this may be due to lack of stress as plants under pathogen stress encourage and depend on AMF colonization. Grapevines not under pathogen stress would have less need for AMF symbionts. In contrast to the results observed with rootstocks 101-14 and 5C, none of the ‘home’ or ‘away’ AMF communities influenced any of the growth parameters of Schwarzmann vines in this experiment. This could be due to their relative growth rates, where Schwarzmann was seen to be faster in growing than the other two varieties. While AMF are regarded as beneficial fungi that promote vine growth and physiological performance [18,43,44,45], this is not always the case. This confirmed the results of other studies where AMF inoculation did not influence plant growth in either greenhouse or field studies on grapevines [46,47,48].

In this study, the pathogen might have spread between the planting material during the rooting stages of the grapevine canes in the pumice which was supported by the lower disease incidence and severity at 5 cm compared at 0 cm stem base. This indicates that the infection has entered through the cut ends, and potentially during the rooting stage, from a low level of contamination in the rootstock material spreading under the warm, moist conditions. To prevent this cross-contamination the application of non-specific measures such as hot water treatment (HWT) of cuttings has been suggested by several studies [6,49,50]. Bleach et al. (2013) [51] showed that treatment of dormant nursery grapevine material at 48.5 °C for 30 min reduced black foot disease incidence. Future studies could hot-water treat the rootstock cuttings to reduce the background pathogen level. However, in the current study the effect of AMF inoculation on the black foot disease incidence and severity in vines already infected (non-pathogen inoculated treatments) as well as to protect vines from further infection when subsequently challenged could be determined. A recent study also showed that HWT could have a negative effect on vines by increasing the susceptibility of grapevines when subsequently challenged with trunk pathogens [52]. This was explained by the fact that grapevine cuttings grown in cool climates such as in Australia and New Zealand are more susceptible to injury in HWT than cuttings grown in warm climates [53,54,55].

In this current study, the *Dactylonectria* and *Ilyonectria* spp. mixed isolate inoculum was shown to be pathogenic, infecting three young grapevine rootstocks (101-14, 5C and Schwarzmann) originating from rooted one-year old canes where the roots had been wounded. This confirmed the findings of Probst et al. (2019) [8] where *I. liriodendri* and *D. macrodidyma* were also pathogenic causing high disease incidence and severity in 101-14 and 5C rootstocks. Overall, the disease incidence and severity were, as expected, higher in the pathogen inoculated vines compared with the uninoculated vines. However, isolates identified as *Dactylonectria* and *Ilyonectria* spp. were also isolated from the vines not inoculated with the black foot pathogens. The vines were hand watered which could have resulted in cross-contamination between adjacent pots via water splash dispersal of inoculum. This has been reported in other studies where overhead watering and keeping the soil surface bare favored the dispersal of grapevine trunk pathogen inoculum [8,56]. Although this cannot be discounted, it is more likely that the rootstock cuttings obtained from the commercial nursery were already infected with these pathogens. Sequencing of representatives of the isolates recovered from the vines across all treatments showed these were species not used in the inoculation, such as *D. torrensis*, *D. novozelandica* and *Ilyonectria* sp. The majority of the *Dactylonectria* and *Ilyonectria* species (*D. torrensis*, *D. novozelandica* and *Ilyonectria* sp.) identified as potentially being present in the rootstocks prior to pathogen inoculum have previously both been recorded in New Zealand on grapes [57] and have been reported to be pathogenic to grapevines [1,4].

In New Zealand, there is evidence that rootstock 101-14 may be more susceptible to black foot disease than other rootstocks [58]. In the study of Jaspers et al. (2007) [59], 101-14 was shown to have the highest susceptibility to a mixed inoculum of ‘*Cylindrocarpon*’ spp. with Schwarzmann and 5C having the lowest susceptibility. Probst et al. (2019) [8] however reported that although there was a trend in some assessments for slightly higher disease levels in 101-14 than in 5C, overall, there was no significant difference between the two rootstocks. In the Bleach et al. (2021) [60] study, at higher disease pressure, 101-14 and Schwarzmann were shown to be more susceptible than 5C. This was not the case in this study, where Schwarzmann and 5C were the most susceptible and 101-14 the least based on the disease incidence and severity results. The presence of high disease level could explain some of the differences seen in this study compared with other studies. This might also be related to the genera/species used (either in inoculation or in vineyard soil experiments) which may have affected the relative susceptibility ranking of the rootstocks. This was seen in Brown et al. (2013) [20] study where although all the studied rootstocks were susceptible to *Cylindrocladiella parva* isolates, Riparia Gloire followed by Schwarzmann and 5C were the most susceptible having the highest disease incidence and severity compared to 101-14, being the lowest. Another study demonstrated that both Schwarzmann and Riparia Gloire rootstocks inoculated with *D. pauciseptata* showed similar levels of infection at the assessed times [61].

This study showed that disease incidence/severity was not associated with the decrease in growth parameters as seen in other studies [8] and could indicate that the experimental period was too short. Whitelaw-Weckert (2007) [62] reported that disease development was slow with no above-ground symptoms observed after 18 months, and rotten roots seen only after three years. While this could indicate that glasshouse is a protective environment, this was not the case in this study as disease symptoms were observed after five months of inoculating the vines with the pathogen. 

Of the species used to inoculate the vines, both *Ilyonectria liriodendri* and *Dactylonectria macrodidyma* were recovered from the vines indicating their pathogenicity under the experimental conditions. Isolates identified as *I. europaea* were not identified but this does not necessarily mean that the isolate of *I. europaea* used in the mixed isolate inoculum did not infect the vines as only a representation of the recovered isolates were sequenced for identification. Further, the inoculum level of this isolate was low in the overall inoculum, being only 10^4^ spores/mL, which might have resulted in low levels of infection by this isolate. As well as *Ilyonectria* and *Dactylonectria* spp. other potentially beneficial fungi (*Trichoderma* sp.) and fungi associated with grapevine trunk diseases (*Botryosphaeria* sp., *Diplodia* sp. and *Diporthe* sp.) were recovered from the grapevine plants across the different treatments. The reason why 101-14 rootstock overall had lower black foot disease incidence and severity compared to other rootstocks may have been due to the high proportion of *Fusarium* spp. and *Trichoderma* spp. recovered from this rootstock for most treatments. This, along with AMF inoculation, may have reduced the infection by the black foot pathogen. Studies have shown that AMF and *Trichoderma* spp. have successfully reduced black foot infections in nurseries [9,40,49]. Moreover, some beneficial *Fusarium* spp. are also known to provide plants protection from root pathogens and reduce disease infection in various horticultural plants and tree species [63]. As for rootstocks 5C and Schwarzmann, it was shown the plants were dominated by black foot and other grapevine trunk disease pathogens which could have resulted in the higher level of disease incidence and severity. 

## 5. Conclusions

This study showed that the high level of disease present in the rootstocks limited the effect of AMF community with only small evidence that AMF treatments lowered disease incidence and severity in vines. It was evident that the rootstocks differ in their susceptibility and their interaction with the pathogen. It was also noticed that the high disease incidence and severity did not reduce growth in vines with AMF inoculation compared to the vines inoculated with the pathogen only. This work aimed to fill a gap in knowledge regarding AMF–plant–pathogen interactions but further research is required to understand how the presence of AMF could also increase grapevine growth parameters while vines are severely infected with black foot disease. Moreover, future work in a vineyard site with natural inoculum would be a useful next step. 

## Figures and Tables

**Table 1:** Inoculation treatments applied to three grapevine rootstocks. The fungal inoculants consisted of “home” and “away” AMF communities, and black foot pathogen mixture. Treatment codes are as follows: Ctrl = no microbial inoculation, AMF = Arbuscular mycorrhizal fungi inoculation, Pathogen = black foot inoculation. The pathogen treatment was inoculated both at planting (December) and three months later in March.

Rootstocks	Treatment	AMF Inoculation (November)	Pathogen Inoculation (December/March)	Description
101-14, 5C and Schwarzmann	Ctrl/Ctrl	None	None	No mycorrhizal or pathogen inoculation
101-14, 5C and Schwarzmann	AMF/Ctrl	101-14 AMF	None	Rootstocks pre-inoculated with 101-14 AMF community, but no pathogen inoculation
101-14, 5C and Schwarzmann	AMF/Ctrl	5C AMF	None	Rootstocks pre-inoculated with 5C AMF community, but no pathogen inoculation
101-14, 5C and Schwarzmann	AMF/Ctrl	Schwarzmann AMF	None	Rootstocks pre-inoculated with Schwarzmann AMF community, but no pathogen inoculation
101-14, 5C and Schwarzmann	AMF/Pathogen	101-14 AMF	Pathogen	Rootstocks pre-inoculated with 101-14 AMF community in November and then inoculated with the pathogen in December & March
101-14, 5C and Schwarzmann	AMF/Pathogen	5C AMF	Pathogen	Rootstocks pre-inoculated with 5C AMF community in November and then inoculated with the pathogen in December & March
101-14, 5C and Schwarzmann	AMF/Pathogen	Schwarzmann AMF	Pathogen	Rootstocks pre-inoculated with Schwarzmann AMF community in November and then inoculated with the pathogen in December & March
101-14, 5C and Schwarzmann	Ctrl/Pathogen	None	Pathogen	No mycorrhizal pre-inoculation but the rootstocks inoculated with the pathogen in December & March

## Data Availability

Not applicable.

## Supplementary Materials

The following supporting information can be downloaded at: https://www.mdpi.com/article/10.3390/jof8030250/s1, Figure S1: Colony morphology of representatives isolates of the fungal groups isolated from grapevine stem pieces after 7 days growth on PDA at 20 °C. A: *Trichoderma* sp. B: *Fusarium* sp. C. *Diplodia* sp. D. *Epicoccum* sp. E. *Diaporthe* sp. F. *Botryosphaeria* sp.; Table S1: Morphological and molecular identification of representative of the different fungal morphotypes recovered from the grapevines stem pieces based on sequencing of the ITS region.; Table S2: Sequencing analysis results of the sub-cultured (10%) colonies for pathogen confirmation and identification based on the histone H3 gene region.
