# Supplementary material for: AMF Community Diversity Promotes Grapevine Growth Parameters under High Black Foot Disease Pressure
Source: J Fungi (Basel). 2022 Mar 1;8(3):250. doi: 10.3390/jof8030250 (PMC8950140; doi:10.3390/jof8030250)
Supplement: Supplementary file 1 [file jof-08-00250-s001.zip › jof-1592105-supplementary.pdf]

Supplementary information

Molecular confirmation of identity of other isolated fungal

The identity of representatives of other fungal colonies (20%) commonly recovered from the grapevine stem pieces from the different treatments was determined by sequencing the internal transcribed spacer (ITS) of the rRNA gene (White et al. 1990). The DNA for representative colonies of each morphotype recovered from the stem sections was extracted as previously described in Section 4.2.8.3. The PCR amplification was performed using 10 µL of Thermo Scientific DreamTaq Green PCR Master Mix (2X), 10 pmol of each primer and 7 µL nuclease free water. An initial denaturation and enzyme activation step of 5 min at 95°C was followed by amplification for 40 cycles at the following conditions: 30 s at 95°C, 40 s at 60°C, 40 s at 72°C and a final elongation at 72°C for 10 min. Primers used were ITS1 (5'-TCCGTAGGTGAACCTGCGG-3') and ITS4 (5'-TCCTCCGCTTATTGATATGC-3') (White et al. 1990).

Molecular identification of other fungal groups isolated

The colony morphology description and the molecular identification of representatives of the sub-cultured fungal groups isolated from grapevine wood pieces grown on PDA are presented in Table S1 and Figure S1. The identified fungi belong to *Trichoderma sp.*, *Fusarium sp.*, *Diplodia sp., Epicoccum sp.*, *Diaporthe* sp. and *Botryosphaeria* sp.

Table S1. Morphological and molecular identification of representative of the different fungal morphotypes recovered from the grapevines stem pieces based on sequencing of the ITS region.

No	Colony morphology	Closest match	Similarity	Accession #
A	Light brown colony colour, green spores, circular green margin	*Trichoderma* sp.	99.84%	MT626718
B	Pinkish colony colour, fluffy white growth and irregular white margin	*Fusarium* sp.	99.63%	MT530067
C	White yellowish colony colour, flat growth, and circular white margin	*Diplodia* sp.	99.47%	MF687190
D	Red to brown colony colour, little raised in nature, irregular brown margin	*Epicoccum* sp.	100%	KX664337
E	White colony colour, black spores, flat growth, and circular white margin	*Diaporthe* sp.	99.31%	HQ533144
F	Black colony colour, fluffy raised black growth, and circular black margin	*Botryosphaeria* sp.	99.83%	MH518159

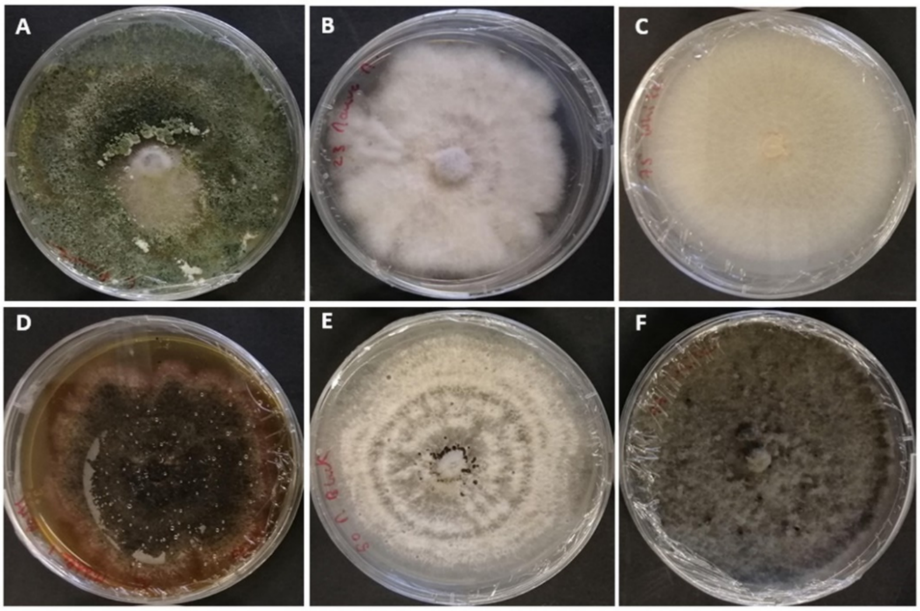

Figure S1. Colony morphology of representatives isolates of the fungal groups isolated from grapevine stem pieces after 7 days growth on PDA at 20°C. A: Trichoderma sp. B: Fusarium sp. C. Diplodia sp. D. Epicoccum sp. E. Diaporthe sp. F. Botryosphaeria sp.

Supplementary information

Table S2. Sequencing analysis results of the sub-cultured (10%) colonies for pathogen confirmation and identification based on the histone H3 gene region.

No	Bp	Closest match	Query cover	Similarity	Accession #
		**101-14 rootstock**			
101-14 AMF	511	*Ilyonectria liriodendri*	99%	97.61%	JF735508
5C AMF	498	*Dactylonectria torresensis*	93%	99.30%	MN561695
Schwarzmann AMF	508	*Ilyonectria liriodendri*	95%	100%	MK330712
101-14 AMF + pathogen	515	*Ilyonectria liriodendri*	96%	98.93%	JF735513
5C AMF + pathogen	506	*Ilyonectria* sp.	92%	99.54%	MH553540
Schwarzmann AMF + pathogen	500	*Dactylonectria novozelandica*	93%	99.78%	MK409915
Pathogen	509	*Ilyonectria liriodendri*	96%	99.72%	MK579278
Control	522	*Ilyonectria liriodendri*	93%	99.12%	JF735513
		**5C rootstock**			
5C AMF	500	*Ilyonectria* sp.	96%	98.94%	MH553540
101-14 AMF	495	*Ilyonectria* sp.	95%	97.56%	MF350439
Schwarzmann AMF	506	*Ilyonectria* sp.	96%	99.20%	MH553540
5C AMF + pathogen	519	*Ilyonectria liriodendri*	96%	99.16%	JF735513
101-14 AMF + pathogen	510	*Ilyonectria liriodendri*	96%	98.95%	MK579278
Schwarzmann AMF + pathogen	510	*Ilyonectria liriodendri*	96%	98.50%	MK330712
Pathogen	485	*Ilyonectria liriodendri*	98%	99.14%	MK579278
Control	489	*Ilyonectria liriodendri*	97%	99.36%	MK579278
		**Schwarzmann rootstock**			
Schwarzmann AMF	483	*Ilyonectria liriodendri*	96%	99.14%	MK579278
5C AMF	495	*Ilyonectria liriodendri*	94%	99.14%	MK330712
101-14 AMF	498	*Dactylonectria torresensis*	91%	99.31%	MN561695
Schwarzmann AMF + pathogen	484	*Dactylonectria macrodidyma*	97%	99.11%	KF633159
5C AMF + pathogen	481	*Dactylonectria macrodidyma*	98%	99.34%	KF633159
101-14 AMF + pathogen	493	*Ilyonectria liriodendri*	98%	98.71%	MK579278
Pathogen	500	*Ilyonectria liriodendri*	96%	98.52%	MK330712
Control	494	*Dactylonectria macrodidyma*	98%	98.29%	KF633159
